# Mapping the aging kidney: a bibliometric atlas of cellular senescence and future frontiers since the 20th century

**DOI:** 10.3389/fneph.2026.1905354

**Published:** 2026-10-07

**Authors:** Jieyan Wang, Mengning Yang, Yifei Li, Yantong Wan, Xiaoying Zhan, Hui Liang

**Affiliations:** 1 Jinglong Jianshe Road, Department of Urology, People’s Hospital of Longhua, Shenzhen, Guangdong, China; 2 School of Rehabilitation Sciences, Southern Medical University, Guangzhou, China; 3 School of Stomatology, Southern Medical University, Guangzhou, Guangdong, China; 4 State Key Laboratory of Cellular Stress Biology, School of Life Science, Faculty of Medicine and Life Sciences, Xiamen University, Xiamen, China; 5 The Third Affiliated Hospital, Southern Medical University, Guangzhou, China

**Keywords:** bibliometric analysis, cellular senescence, chronic kidney disease, ferroptosis, mitochondrial dysfunction

## Abstract

**Background:**

Kidney disease is a major global public health challenge. The pathological mechanisms linking chronic kidney disease (CKD) and age-related kidney function decline are complex. Cellular senescence and its senescence-associated secretory phenotype (SASP) act as key links between kidney aging and disease progression. Although publications in this field have surged, a systematic bibliometric analysis is needed to outline its knowledge framework, research hotspots, and future trends. This study aims to quantitatively analyze global publication trends, core contributors (countries, institutions, journals, and authors), and cooperation networks to map the dynamic evolution of this field.

**Methods:**

We conducted a comprehensive literature search across both the Web of Science Core Collection (WoSCC) and Scopus databases to retrieve original articles and reviews concerning cellular senescence and kidney aging published between 2000 and 2025. After merging the datasets and removing duplicates, a meticulous bibliometric analysis was executed using Excel 365, CiteSpace, VOSviewer, and Bibliometrix (an R package) to map the knowledge framework and cooperation networks.

**Results:**

This analysis included 2,191 relevant papers involving 16,545 researchers. The overall publication volume showed significant growth, reaching a peak in 2025. The United States leads the field with the most publications and the highest citation frequency. China ranks second in publications but fourth in citations, reflecting a gap in research quality. The collaboration network indicates that the United States is the core node, maintaining close ties with countries like China and Germany. The *American Journal of Physiology-Renal Physiology* ranked first in publication volume, while the *Journal of the American Society of Nephrology* achieved the highest citation frequency. Xiang-Mei Chen and Anette Melk were identified as core contributing scholars. Keyword analysis established “aging” (825 times) and “cellular senescence” (330 times) as the central concepts. Emerging topics such as “machine learning,” “ferroptosis,” and “Mendelian randomization” are rapidly rising as research hotspots.

**Conclusion:**

This study maps the evolutionary trends of research on cellular senescence and related kidney diseases, identifying current frontiers and providing data to support future research. The rise of concepts like machine learning, ferroptosis, and Mendelian randomization marks a deep shift in the research paradigm, offering new perspectives for future scientific exploration.

## 1 Introduction

With the acceleration of global population aging, age-related diseases pose severe public health challenges (1, 2). The kidney exhibits marked structural and functional vulnerability during aging, characterized by glomerulosclerosis, tubular atrophy, interstitial fibrosis, and vascular rarefaction (3, 4). These progressive changes compromise renal functional reserve and significantly increase susceptibility to acute kidney injury (AKI) and chronic kidney disease (CKD), while CKD itself is recognized as a clinical state of accelerated biological aging (5, 6). Cellular senescence—defined by irreversible cell cycle arrest and persistent metabolic activity—serves as the central mechanistic link and primary driver of renal aging and disease progression (7, 8). Chronic physiological aging and persistent pathological stresses induce premature senescence in key renal parenchymal populations, including tubular epithelial cells, podocytes, and vascular endothelial cells (9, 10). These senescent cells acquire a senescence-associated secretory phenotype (SASP), releasing pro-inflammatory cytokines, chemokines, and profibrotic factors that trigger paracrine senescence in neighboring healthy cells, disrupt regenerative tissue repair, and sustain sterile microinflammation (11, 12). This ongoing cascade fuels extracellular matrix accumulation, nephron loss, and renal architectural remodeling, establishing a self-amplifying cycle between cellular senescence and whole-organ renal decline (13). Consequently, selectively eliminating senescent cells via senolytics or modulating SASP components has emerged as a promising therapeutic strategy to retard renal aging and mitigate CKD progression (14, 15).

Despite significant progress over the past two decades, research in this field faces prominent limitations. Existing investigations remain fragmented, characterized by a substantial disconnect between microscopic molecular pathways (such as ferroptosis, mitochondrial quality control defects, impaired autophagy, and DNA damage response) and macroscopic clinical phenotypes (such as diabetic kidney disease, hypertensive nephrosclerosis, and allograft senescence) (16, 17). While emerging non-apoptotic death mechanisms like ferroptosis have shifted the research focus toward the intersection of metabolic regulation and cell fate determination (18–20), the global network linking these subcellular perturbations to clinical renal outcomes remains inadequately integrated. Moreover, current literature is predominantly limited to qualitative narrative summaries of isolated signaling pathways, lacking macro-level, quantitative synthesis to map the overarching intellectual structure, global collaborative patterns, and dynamic evolutionary trajectories across the discipline (21). Bibliometrics offers an objective, reproducible approach using co-citation networks and keyword clustering to uncover latent knowledge structures, author networks, and evolutionary frontiers within complex medical domains (22). Therefore, a systematic and visualized bibliometric analysis is urgently needed to bridge these gaps. Utilizing records retrieved from the Web of Science Core Collection and Scopus databases spanning 2000 to 2025, this study systematically analyzes global publication trends, core contributors, international collaboration networks, and shifting mechanistic paradigms. By identifying critical research bottlenecks and emerging hotspots, this work aims to establish a comprehensive knowledge atlas and provide data-driven strategic direction for future mechanistic exploration and clinical translation.

## 2 Materials and methods

### 2.1 Data sources and search strategy

The literature matrix for this study was constructed via a synchronized bibliometric retrieval spanning two primary source repositories: the Web of Science Core Collection (WoSCC, specifically searching the Science Citation Index Expanded, SCIE) and Scopus. The temporal sampling window was restricted from 2000 to 2025, capturing peer-reviewed, English-language original articles and reviews. The literature searches across both databases were executed simultaneously on January 15, 2026, to prevent discrepancies caused by daily database updates. To minimize selection bias and comprehensively delineate the thematic landscape, advanced semantic field search queries were formulated using matching Boolean strings encompassing renal pathology and cell senescence biomarkers. The precise programmatic search algorithms utilized for each database were structured as follows:WoSCC Query Syntax: TI=((kidney OR renal OR glomerulonephritis OR nephropathy) AND (senescence OR senescent OR “aging cell*” OR “senescence-associated” OR “SASP” OR “aging” OR “age-related”)) OR AK=((kidney OR renal OR glomerulonephritis OR nephropathy) AND (senescence OR senescent OR “aging cell*” OR “senescence-associated” OR “SASP” OR “aging” OR “age-related”)). Scopus Query Syntax: (TITLE((kidney OR renal OR glomerulonephritis OR nephropathy) AND (senescence OR senescent OR “aging cell*” OR “senescence-associated” OR “SASP” OR “aging” OR “age-related”)) OR KEY((kidney OR renal OR glomerulonephritis OR nephropathy) AND (senescence OR senescent OR “aging cell*” OR “senescence-associated” OR “SASP” OR “aging” OR “age-related”))) AND (LIMIT-TO(DOCTYPE, “ar”) OR LIMIT-TO(DOCTYPE, “re”)) AND (LIMIT-TO(LANGUAGE, “English”)).

### 2.2 Data collection and analysis

The conceptual eligibility framework strictly restricted inclusion to peer-reviewed, English-language original research articles and comprehensive reviews published within the designated 2000–2025 timeframe. From an initial pool of 3,906 records retrieved from the Web of Science Core Collection (n = 1,742) and Scopus (n = 2,164), 15 non-English language documents and 171 other document modalities—including conference proceedings, books, book chapters, editorials, short communications, and letters—were systematically excluded, leaving 2,191 records eligible for full-text assessment (**Figure 1**). To ensure substantive programmatic and thematic relevance to the intersection of renal pathophysiology and cellular senescence, a dual-reviewer independent screening protocol was implemented. Two independent reviewers meticulously evaluated the titles, keywords, and abstracts of the retrieved records to disqualify irrelevant publications. Any discrepancies regarding document eligibility were resolved through thorough discussion until a consensus was reached. Following active filtration, the finalized citation matrices were exported in plain-text format containing full records and cited references (specifically utilizing the “Full Record and Cited References” plain-text format for WoSCC and standardized tagged bibliographic text for Scopus). To construct a unified bibliometric corpus, records exported from both databases were integrated and deduplicated using a custom R-based preprocessing pipeline. All records were imported in plain-text format and merged into a standardized dataset while preserving the original tagged bibliographic structure. Only valid entries beginning with the publication-type field (PT) were retained for downstream analyses. Duplicate removal was performed using a hierarchical strategy based primarily on Digital Object Identifier (DOI) matching and secondarily on publication title matching. DOI information (DI field) was first extracted and normalized by converting characters to lowercase and removing redundant whitespace. Records sharing identical normalized DOIs were collapsed into a single entry. For records lacking DOIs, duplicate detection was subsequently conducted using publication titles (TI field) by normalizing case and compressing irregular spacing. Entries sharing identical normalized titles were regarded as duplicates and removed. In instances of metadata discrepancies between WoSCC and Scopus (such as author name initials and journal abbreviations), the WoSCC metadata schema was prioritized for standardization. The extracted metadata matrices (publication years, geographic origins, corporate affiliations, source journals, co-authorship networks, and structural keywords) were analyzed using specialized computational tools. Microsoft Excel 2016 was used to track annual publication and citation trends. CiteSpace (Version 6.3.R1) was deployed to standardize knowledge units, quantify similarities, and conduct reference co-citation clustering as well as citation-burst detection. CiteSpace parameters were set as follows: Link Retaining Factor (LRF) = 3.0, Maximum Links Per Node (L/N) = 10, Look Back Years (LBY) = 5, Percentage of Nodes to Label = 1.0%, and Threshold (k) = 25. Cluster quality was evaluated using modularity (Q) and silhouette (S) values, with Q > 0.5 and S > 0.7 indicating satisfactory clustering. VOSviewer (Version 1.6.20) was employed to construct network maps emphasizing structural logic and collaboration patterns. For VOSviewer analyses, minimum thresholds were set as follows: 8 occurrences for keywords, 5 publications for authors, 50 co-citations for co-cited authors, 175 co-citations for co-cited journals, 10 publications for countries/regions, and 7 publications for journals. In the network maps, node size represented publication, occurrence, or co-citation frequency, and link thickness indicated the strength of relationships. Finally, Bibliometrix (an R package) was utilized to model thematic evolution, Multiple Correspondence Analysis (MCA), and keyword dynamics.

**Figure 1:**
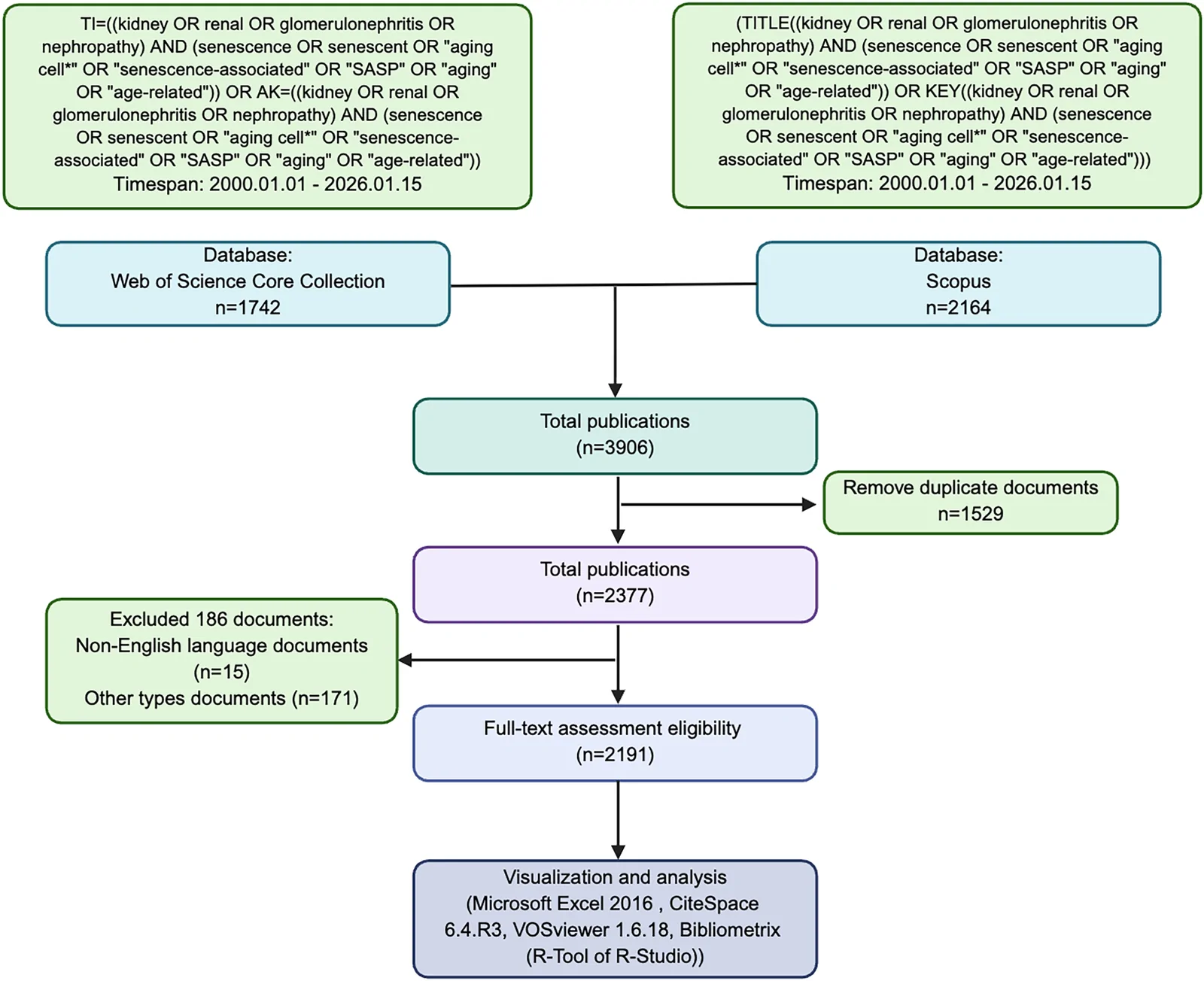
Flowchart of the literature search, screening, and selection process for the bibliometric analysis.

## 3 Results

### 3.1 Evolution of annual research output

Through a systematic search of the WoSCC and Scopus database, this study included 2,191 papers on kidney aging published between 2000 and 2025. As shown in **Figure 2**, the annual output grew in distinct stages. During the “initial exploration stage” from 2000 to 2018, annual publications remained below 100, reflecting a period of basic knowledge accumulation. Since 2018, the research scale entered a “rapid expansion stage,” surpassing 100 annual publications and reaching a peak of 320 papers in 2025. Academic impact surged simultaneously, with total annual citations reaching a high of 8,577 in 2025. This synergistic growth in both publication output and citations not only demonstrates a globally surging interest in kidney aging mechanisms, but also signals that the field has evolved from early descriptive studies into an accelerated phase of breakthrough discoveries.

**Figure 2:**
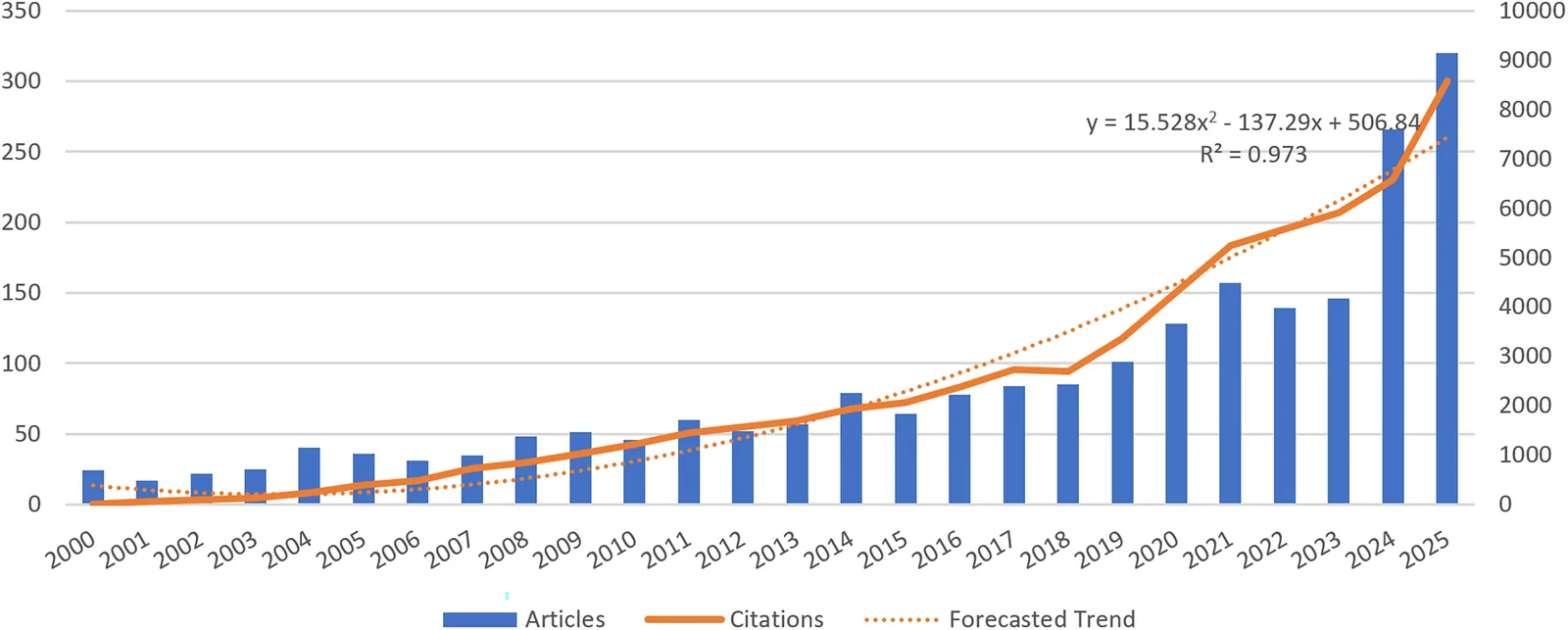
Global publication and citation trends of kidney and cellular senescence research from 2000 to 2025.

### 3.2 Country analysis

As shown in **Table 1**, the spatial distribution of research output reveals a highly uneven global landscape. The United States firmly holds the top global position with 587 papers... The global collaboration network (**Figure 3A**) further reveals cross-border interaction patterns: circle circumferences correspond to national publication volumes, while line thickness indicates the depth of collaboration. Analysis shows that US-China collaboration is currently the strongest global pathway, followed by US-South Korea collaboration. Regarding publication models (**Figure 3B**), the US leads with over 400 corresponding authors, closely followed by China with nearly 400. In terms of international cooperation breadth, aside from Sweden, which exhibits a significant trend toward Multiple Country Publications (MCP), other high-output countries predominantly rely on Single Country Publications (SCP). This reflects that research resources in the kidney aging field remain strongly regionally concentrated. Furthermore, although Germany does not rank first in publication volume, it ranks second globally in Total Link Strength, highlighting its critical hub role in connecting the global research network.

**Figure 3:**
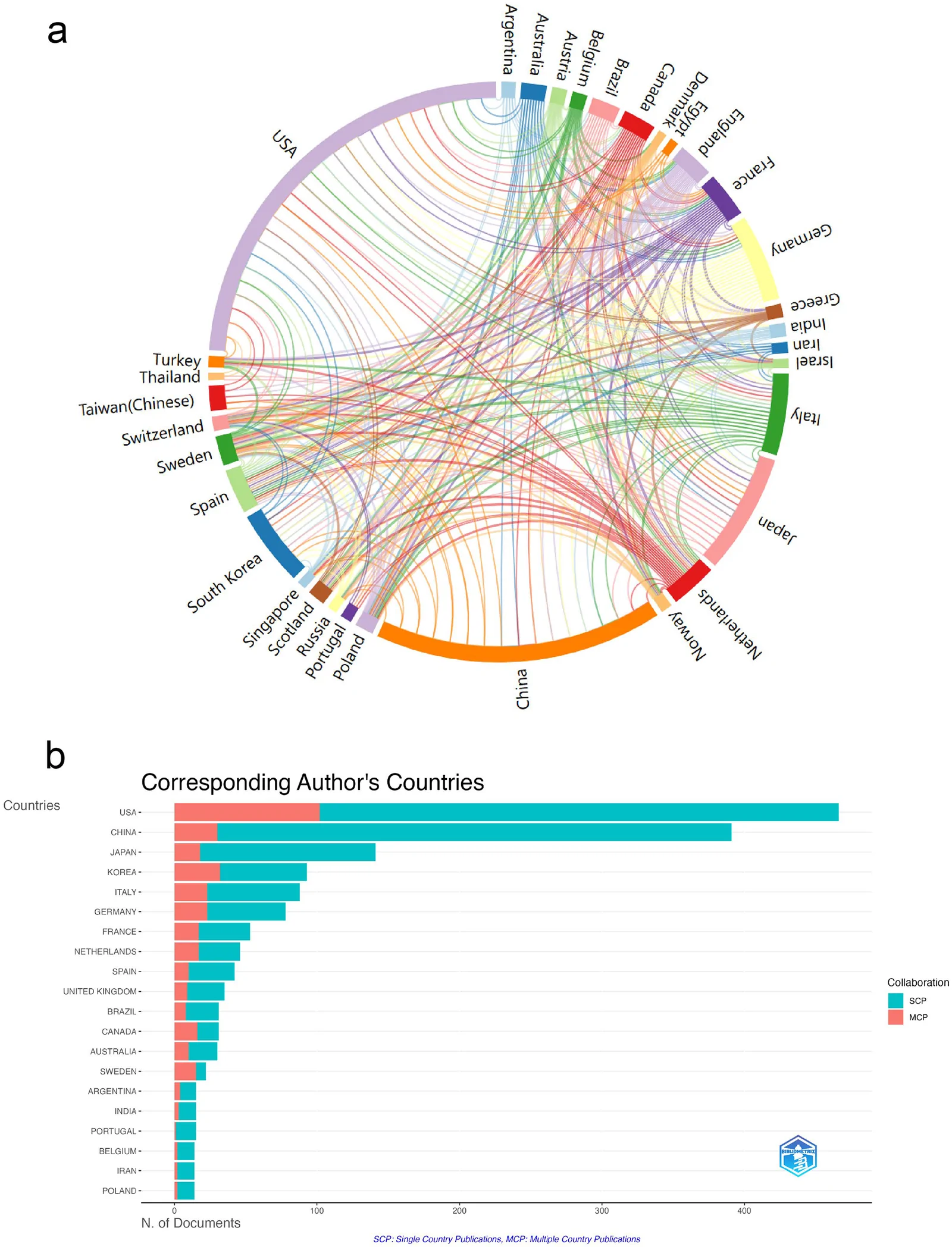
Global research landscape, academic impact, and collaborative networks in kidney aging research. **(A)** National output and academic impact. Node size reflects publication volume, highlighting the US-China leadership and the significant disparity between output quantity and citation quality. **(B)** Publication models and authorship. Displays corresponding author distribution, the prevalence of Single Country Publications (SCP) and Multiple Country Publications (MCP).

**Table 1:** Top10 countries/regions in terms of number of articles published and number of citations accordingly.

Rank	Countries	Documents	Countries	Citations	Countries	Total link strength
1	USA	587	USA	25229	USA	258
2	China	399	China	7734	Germany	117
3	Japan	166	Germany	5185	Italy	91
4	Germany	120	Japan	5118	China	87
5	Italy	113	France	3064	Netherlands	79
6	South Korea	105	Italy	2876	Sweden	73
7	Netherlands	65	Netherlands	2569	Spain	66
8	France	63	South Korea	2412	England	61
9	Spain	62	Canada	2248	Japan	59
10	England	50	Spain	1792	France	57

### 3.3 Institution analysis

The distribution of research output across institutions further delineates the global academic landscape of the kidney aging field. **Table 2** reveals that the Mayo Clinic leads globally with 44 publications, closely followed by the Chinese People’s Liberation Army (PLA) General Hospital with 38. In terms of academic impact, the Mayo Clinic demonstrated the highest authority with 3,651 total citations, significantly outperforming the second and third-ranked University of Pittsburgh (1,901 citations) and University of California, San Francisco (UCSF) (1,788 citations). Notably, 60% of the top ten institutions by publication volume and six of the top ten by citation frequency are located in the United States. This strongly highlights the overarching dominance of the US in kidney aging research.

**Table 2:** Top 10 institutions in the field of kidney and cellular senescence research.

Rank	Institution	Publications	Original country	Institution	Citations	Original country
1	Mayo Clin	44	United States	Mayo Clin	3651	United States
2	Chinese Peoples Liberat Army Gen Hosp	38	China	Univ Pittsburgh	1901	United States
3	Southern Med Univ	37	China	Univ Calif San Francisco	1788	United States
4	Univ Calif San Francisco	33	United States	NIA	1723	United States
5	NIA	32	United States	Univ Calif Los Angeles	1410	United States
6	Pusan Natl Univ	32	South Korea	Chinese Peoples Liberat Army Gen Hosp	1295	China
7	Univ Pittsburgh	32	United States	Hannover Med Sch	1284	Germany
8	Univ Washington	31	United States	Univ Washington	1226	United States
9	China Med Univ	29	China	Karolinska Inst	1152	Sweden
10	Johns Hopkins Univ	25	United States	Univ Alberta	1082	Canada

VOSviewer clustering analysis indicates that institutional collaboration is highly clustered. As shown in **Figure 4A**, global research forces are divided into nine core clusters, led by key institutions such as Southern Medical University, the PLA General Hospital, Johns Hopkins University, Hannover Medical School, UCSF, and the University of Pittsburgh. This distribution pattern not only reveals the regional characteristics of research groups but also provides data-driven support for identifying potential global collaboration opportunities. Furthermore, a timeline analysis of research evolution (**Figure 4B**) shows distinct periods of activity among institutions. Harvard University and Wake Forest University entered the field early, establishing the foundational knowledge base for kidney aging research. In contrast, Chinese institutions, represented by Southern Medical University and China Medical University, have experienced a rapid surge in publication volume in recent years. This significant temporal shift—from early Western dominance to recent Chinese activity—indicates that emerging research forces, led by China, are accelerating their engagement in this field and gradually becoming key drivers of future frontier advancements.

**Figure 4:**
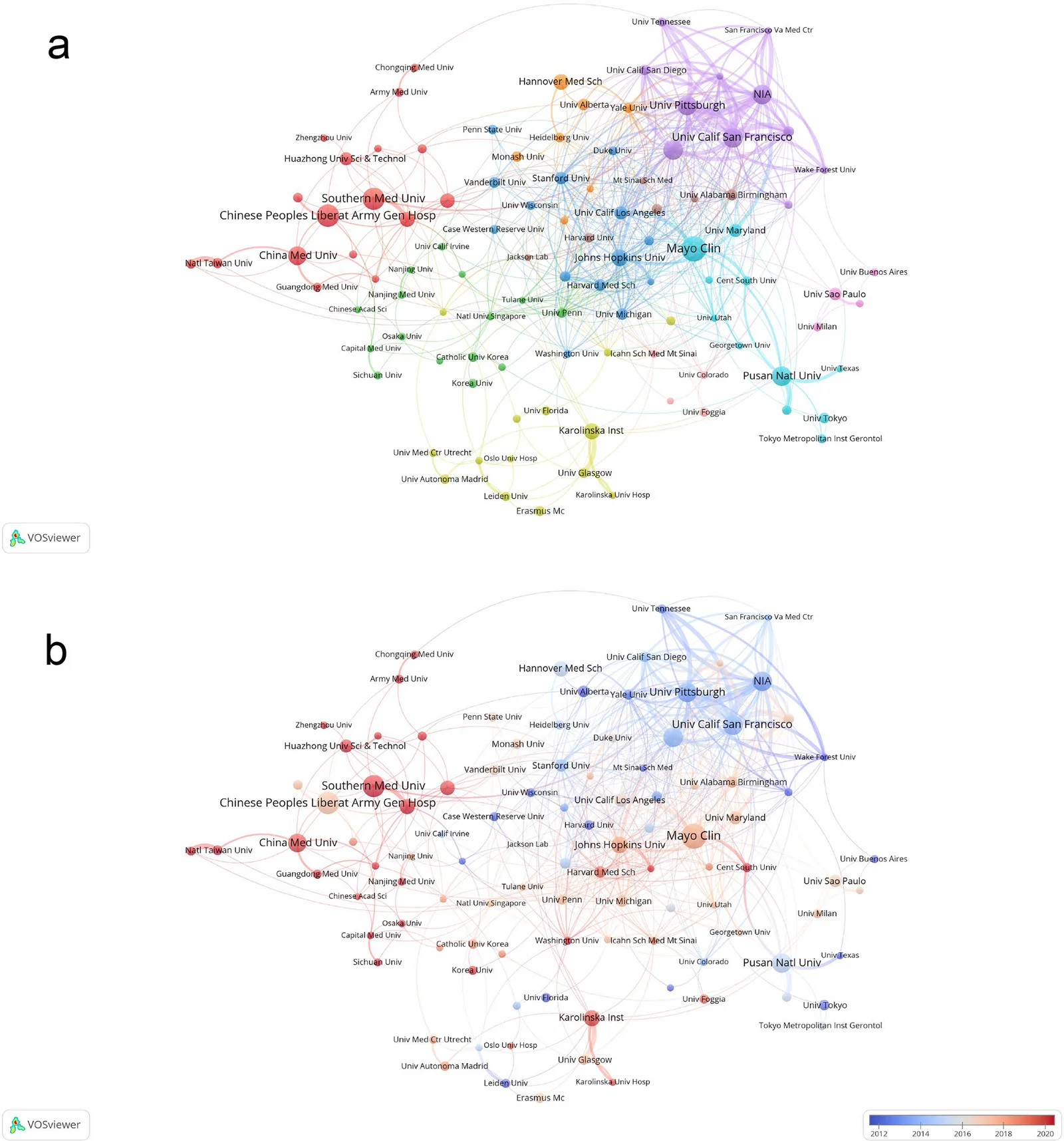
Institutional collaborative networks and temporal evolution in kidney aging research. **(A)** Institutional co-authorship network: Node size maps to the volume of institutional publications, and the links represent collaborative relationships between institutions. **(B)** Timeline visualization: The color gradient shifting from blue to red designates the chronological sequence of publication dates from early to recent.

### 3.4 Journal analysis

Through visualized analysis, this study systematically evaluated the productivity and academic impact of journals in the field of kidney aging. Research findings are distributed across 51 academic journals. In terms of publication scale (**Table 3**), the *American Journal of Physiology-Renal Physiology* (50 papers) and *Kidney International* (48 papers) are the most productive carriers, while the *International Journal of Molecular Sciences* (43 papers), *Experimental Gerontology* (42 papers), and *Aging Cell* (33 papers) also demonstrate significant publishing activity. Regarding academic influence and authority, the *Journal of the American Society of Nephrology* (JASN) (4,666 citations) and *Kidney International* (4,629 citations) lead with a dominant advantage, both exceeding 4,000 total citations and establishing their roles as the core knowledge foundation of the field. These are followed by *Am J Physiol-Renal* (1,785), *Am J Kidney Dis* (1,746), and *Nephrol Dial Transpl* (1,676). Using the VOSviewer affinity clustering algorithm, journals were categorized into five primary academic camps, where the yellow cluster focuses on geriatrics and physiological foundations, the red cluster centers on clinical correlations between the kidney and aging, the green cluster specializes in renal immunology, the dark blue cluster covers frontiers in cellular and molecular biology, and the light blue cluster involves drug development and clinical translational research (**Figure 5A**). **Figure 5B** reveals the evolutionary trends of journal associations over time, with node colors reflecting shifts in publication years and identifying the *American Journal of Physiology-Renal Physiology* as a pivotal hub connecting research from 2010 to 2020 with the highest node significance. Furthermore, the dual-map overlay of journals (**Figure 5C**) delineates the pathways of knowledge flow, showing that citing journals are concentrated in molecular biology, immunology, and clinical medicine, while cited journals primarily point to foundational disciplines such as molecular biology, genetics, and health sciences. This positioning highlights the interdisciplinary nature of the field as it permeates from basic theory toward clinical application. Finally, co-citation network analysis (**Figure 5D**) identifies four specialized research directions: a cell biology cluster represented by *Aging Cell*, a nephrology cluster centered on *JASN*, a cardiovascular cluster associated with the circulatory system, and an immunology-focused cluster. This landscape objectively reflects the multi-dimensional academic penetration of kidney aging research within the life sciences.

**Table 3:** Top 10 journals in kidney and cell senescence research by publication and citation counts.

Rank	Journal	Publications	IF(JCR2023)	JCR quartile	Co-Cited-Journal	Citations	IF(JCR2023)	JCR quatile
1	American Journal of Physiology-Renal Physiology	50	4.3	Q1	J Am Soc Nephrol	4666	8.5	Q1
2	Kidney International	48	12.6	Q1	Kidney Int	4629	12.6	Q1
3	International Journal of Molecular Sciences	43	4.9	Q1	Am J Physiol-Renal	1785	3.4	Q1
4	Experimental Gerontology	42	3.3	Q2	Am J Kidney Dis	1746	9.4	Q1
5	Aging Cell	33	8.0	Q1	Nephrol Dial Transpl	1676	4.8	Q1
6	Journals of Gerontology Series A-Biological Sciences and Medical	25	4.3	Q1	Nature	1515	50.5	Q1
7	Aging-Us	23	3.9	Q2	J Clin Invest	1344	13.3	Q1
8	Frontiers in Immunology	23	5.7	Q1	P Natl Acad Sci Usa	1283	9.4	Q1
9	Journal of The American Society of Nephrology	23	8.5	Q1	J Biol Chem	1214	4.0	Q2
10	Nephrology Dialysis Transplantation	19	4.8	Q1	Plos One	1150	2.9	Q2

**Figure 5:**
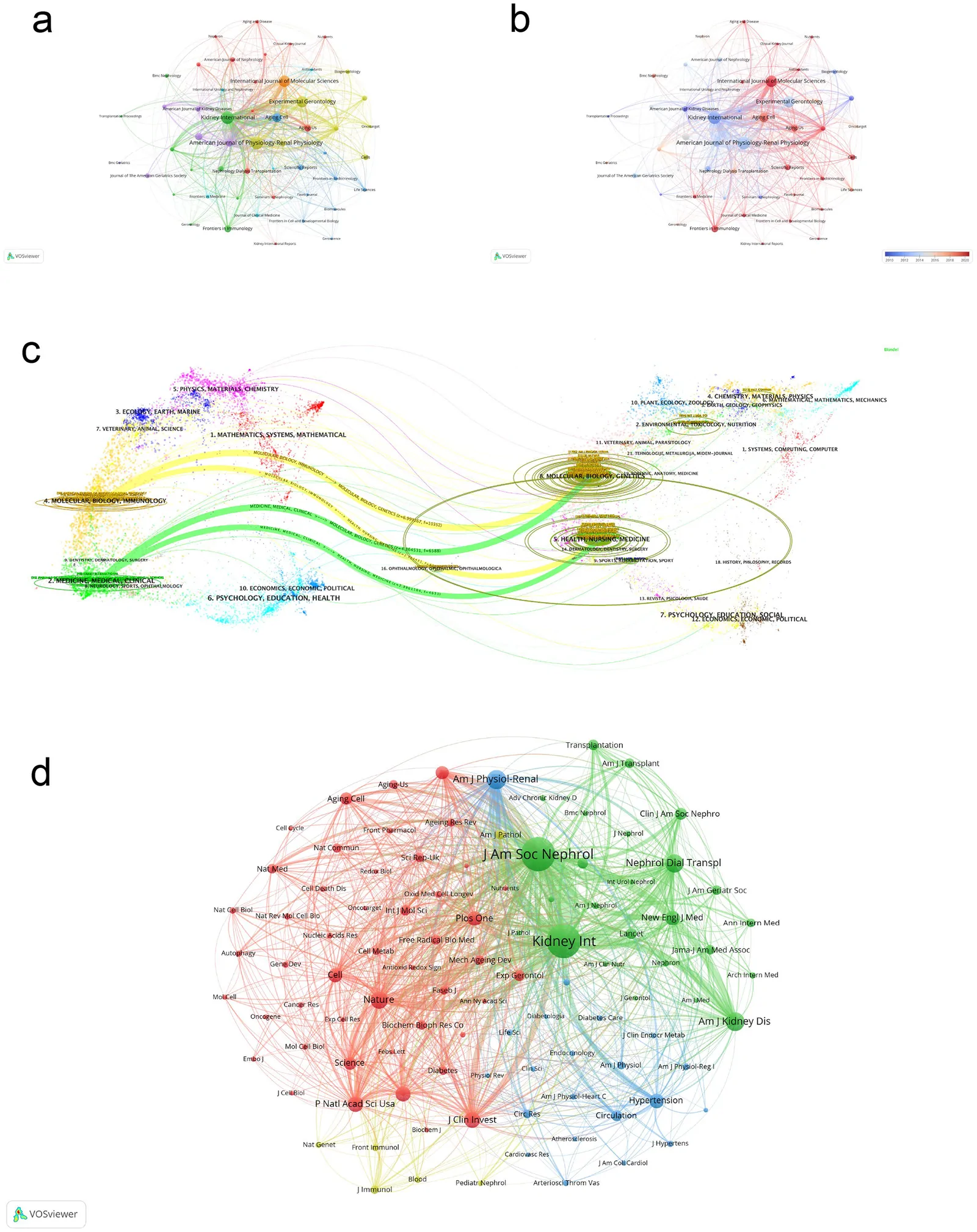
Journal productivity and knowledge flow in kidney aging research. **(A)** Journal clustering network: Related journals are categorized into five distinct academic camps, distinguished by color coding. **(B)** Temporal evolution visualization: The color gradient from blue to red represents the chronological evolution of research, with link thickness indicating the strength of association between journals. **(C)** Dual-map overlay: Labels on the right denote citing journals, while labels on the left denote cited journals; colored curves represent the flow of knowledge from citing to cited sources. **(D)** Co-citation network analysis: Journals are partitioned into four distinct color-coded clusters, each representing a specialized research direction, with links illustrating the interconnections between clusters.

### 3.5 Author analysis

Through bibliometric analysis, this study identified 16,545 scholars dedicated to kidney aging research. According to the author statistics, Xiang-Mei Chen from the PLA General Hospital ranks first in publication volume with 40 papers, establishing her leadership in the field, while Anette Melk leads globally in academic impact with 379 total citations (**Table 4**). The social collaboration network among authors exhibits significant clustering characteristics, as visualized in **Figure 6A**. The map reveals strong intra-cluster cooperation patterns—exemplified by the influential academic partnership between Xiang-Mei Chen and Xuefeng Sun—yet highlights a relative scarcity of cross-disciplinary collaboration between different clusters. Furthermore, **Figure 6B** displays normalized citation indicators to clarify the academic standing of researchers, with Andrew D. Rule and Youhua Liu ranking at the forefront, reflecting their profound scholarly contributions. Co-authorship network analysis (**Figure 6C**) identifies the core hubs of the collaborative system, where A. Melk, A. Denic, and R. Schmitt play pivotal roles in connecting the global research network. Currently, the kidney aging field has formed several mature research clusters centered around scholars such as Xiang-Mei Chen; however, future breakthroughs will likely depend on enhanced cross-cluster and interdisciplinary collaboration to facilitate the flow of knowledge.

**Table 4:** Top 10 authors and co-cited authors in the field of kidney and cell senescence research.

Rank	Author	Documents	Total link strength	Countries/regions	Institution	Author	Co-citations	Total link strength	Countries/regions	Institution
1	Chen, Xiang-Mei	40	106	China	Chinese PLA General Hospital	Melk, A	379	4607	Germany	Hannover Medical School
2	Cai, Guang-Yan	29	88	China	Chinese PLA General Hospital	Levey, As	274	1702	United States	Tufts Medical Center
3	Chung, Hae Young	23	59	South Korea	Pusan National University	Baylis, C	194	1996	United States	University of Florida
4	Melk, Anette	21	31	Germany	Hannover Medical School	Schmitt, R	194	2467	Germany	Hannover Medical School
5	Stenvinkel, Peter	18	27	Sweden	Karolinska Institutet	Lindeman, Rd	193	2402	United States	University of New Mexico
6	Sun, Xuefeng	18	55	China	Chinese PLA General Hospital	Denic, A	182	1940	United States	Mayo Clinic
7	Lerman, Lilach O.	17	69	United States	Mayo Clinic	Rule, Ad	171	2327	United States	Mayo Clinic
8	Rule, Andrew D.	17	32	United States	Mayo Clinic	Coresh, J	164	1467	United States	Johns Hopkins University
9	Schmitt, Roland	16	29	Germany	Hannover Medical School	Stenvinkel, P	155	1616	Sweden	Karolinska Institutet
10	Shlipak, Michael G.	16	63	United States	University of California, San Francisco	Hu, Mc	152	1554	United States	UT Southwestern Medical Center

**Figure 6:**
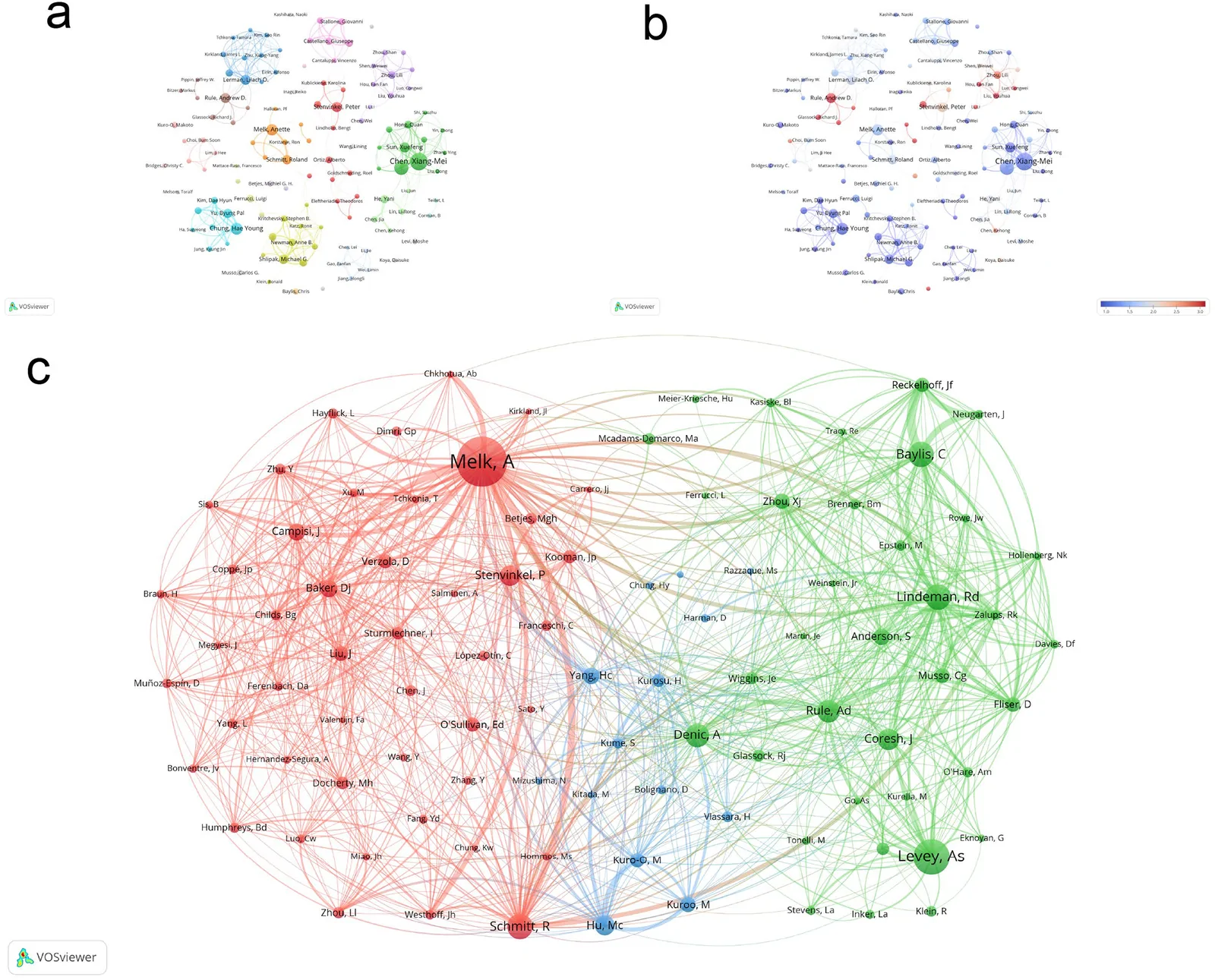
Scholar collaboration, academic impact, and core hubs in kidney aging research. **(A)** Scholar collaboration network: Nodes of different colors represent individual scholars, and links indicate their collaborative relationships. **(B)** Normalized citation indicators: The color gradient shifting from cool to warm maps the normalized citation frequency, with warmer colors indicating higher citation impact. **(C)** Co-authorship network analysis: Links signify co-authorship relations between scholars, illustrating the density and structure of academic cooperation.

In addition, this study employed Lotka’s Law to scientifically evaluate the distribution of author productivity in the field of kidney aging. As illustrated in the attached image, the chart utilizes a log-log coordinate system, where the horizontal axis represents the number of documents written and the vertical axis denotes the proportion of authors at each productivity level. The dashed line represents the theoretical distribution, while the solid line depicts the actual observed data trajectory. The analysis reveals that over 75% of authors in the kidney aging field have published only one article. This phenomenon suggests that the field is currently in a phase of large-scale expansion and rapid initiation, attracting a significant influx of new researchers due to its frontier nature. However, the observed curve shows that the proportion of authors with 2 to 10 publications is significantly lower than the theoretical prediction, reflecting that the number of scholars capable of sustained research output has not yet reached an ideal level. Notably, the proportion of highly productive authors with more than 10 papers is extremely low, indicating a current lack of absolute “core high-output leaders” in the field. In summary, kidney aging research still possesses vast potential for deep exploration and academic breakthroughs, highlighting an urgent need for more researchers with sustained output capabilities to further expand the depth of the discipline.

### 3.6 Reference analysis

Through an in-depth analysis of the reference co-citation network, this study identified 18 knowledge clusters representing distinct research directions. As shown in **Figure 7A**, clustering analysis based on document similarity reveals that “aging” is the largest and most extensively connected core theme, forming the center of the kidney aging research knowledge map. The evolutionary trajectory of research focus over time reveals a transition from clinical manifestations to molecular mechanisms. During the “knowledge accumulation phase”, early research was highly concentrated on cluster #3 chronic allograft nephropathy, before gradually expanding into related disciplines such as #17 transplantation, #14 cardiovascular system, and #10 vessels. As the research entered the “field expansion phase,” the emergence of new clusters like #1 ischemia and #4 evolution marked a profound evolution in research paradigms. Most recently, clusters such as #6 diabetic kidney disease and #7 cell cycle arrest reflect high academic interest in metabolic kidney diseases and the frontiers of cell fate regulation.

**Figure 7:**
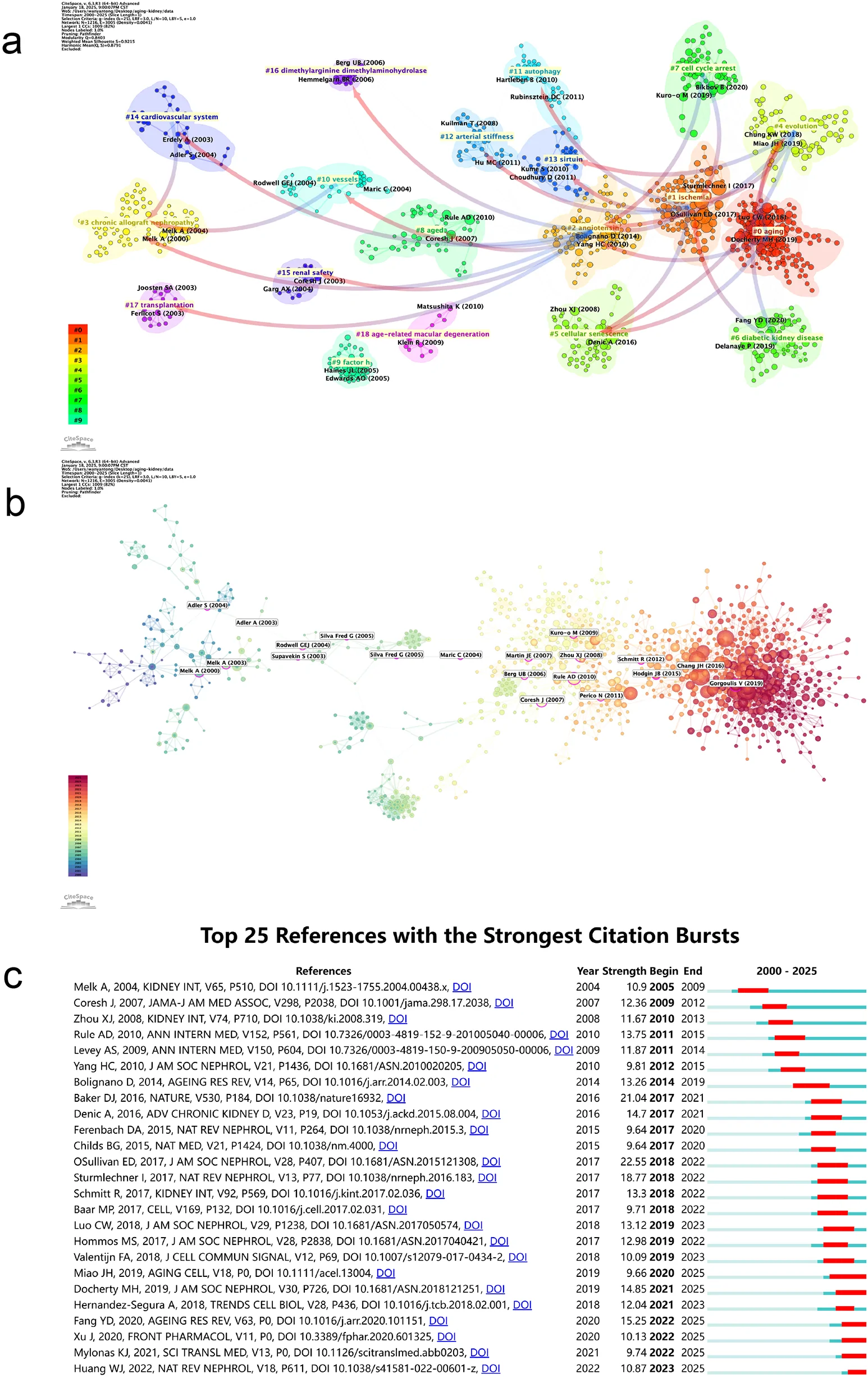
Reference co-citation network, author activity, and citation bursts in kidney aging research. **(A)** Reference co-citation clustering map: illustrates sub-clusters generated based on document similarity, with the connecting edges representing the strength of their association. **(B)** Author activity distribution: a color gradient from cool to warm visually indicates the increasing trend of author activity among the cited references. **(C)** Citation burst analysis: depicts the temporal citation characteristics and evolutionary dynamics of keyword clusters over the analyzed time span.

In terms of citation bursts, several high-impact publications have defined the academic frontier of the field. As shown in the **Table 5** of the top 15 most cited references, the clinical trial report by Hickson LJ et al. on using senolytics (dasatinib plus quercetin) to clear human senescent cells has been cited 758 times, representing one of the most landmark clinical translation studies to date. The author activity distribution map (**Figure 7B**) reflects a generational shift among cited researchers: early research (marked in purple) is represented by Melk A (2000), while the recent research frontier (marked in red) is led by scholars such as Gorgoulis V (2019). **Figure 7C** further lists the 25 references with the strongest citation bursts. The review by O’Sullivan Eoin D (2017) ranks first with a burst strength of 22.55, with its citation surge lasting from 2018 to 2022. Additionally, the study by Darren J. Baker (2013) published in *Nature* regarding p16-positive cells shortening healthspan also demonstrated significant academic influence (Strength = 21.04). Notably, multiple core publications concerning the mechanisms of renal cellular senescence maintain a citation surge into 2025, suggesting that this topic will remain a focal point of discussion for years to come.

**Table 5:** Top 15 most cited articles in the field of kidney and cell senescence research.

Rank	Author	Article title	Source title	Cited	Year	Document type	DOI
1	Hickson, LJ. et al.	Senolytics decrease senescent cells in humans: Preliminary report from a clinical trial of Dasatinib plus Quercetin in individuals with diabetic kidney disease	EBIOMEDICINE	758	2019	Article	10.1016/j.ebiom.2019.08.069
2	Denic, A. et al.	Structural and Functional Changes With the Aging Kidney	ADVANCES IN CHRONIC KIDNEY DISEASE	478	2016	Article	10.1053/j.ackd.2015.08.004
3	Weinstein, JR. et al.	The Aging Kidney: Physiological Changes	ADVANCES IN CHRONIC KIDNEY DISEASE	414	2010	Article	10.1053/j.ackd.2010.05.002
4	Donoso, LA. et al.	The role of inflammation in the pathogenesis of age-related macular degeneration	SURVEY OF OPHTHALMOLOGY	403	2006	Review	10.1016/j.survophthal.2005.12.001
5	Kurella, M. et al.	Chronic kidney disease and cognitive impairment in the elderly: The health, aging, and body composition study	JOURNAL OF THE AMERICAN SOCIETY OF NEPHROLOGY	361	2005	Article	10.1681/ASN.2005010005
6	Verhave, JC. et al.	Estimation of renal function in subjects with normal serum creatinine levels: Influence of age and body mass index	AMERICAN JOURNAL OF KIDNEY DISEASES	337	2005	Article	10.1053/j.ajkd.2005.05.011
7	O’Sullivan, ED. et al.	Renal Aging: Causes and Consequences	JOURNAL OF THE AMERICAN SOCIETY OF NEPHROLOGY	320	2017	Review	10.1681/ASN.2015121308
8	Turnheim, K	When drug therapy gets old: pharmacokinetics and pharmacodynamics in the elderly	EXPERIMENTAL GERONTOLOGY	312	2003	Review	10.1016/S0531-5565(03)00133-5
9	Murray, AM	Cognitive impairment in the aging dialysis and chronic kidney disease populations: An occult burden	ADVANCES IN CHRONIC KIDNEY DISEASE	297	2008	Article	10.1053/j.ackd.2008.01.010
10	Li, SA. et al.	Immunohistochemical localization of Klotho protein in brain, kidney, and reproductive organs of mice	CELL STRUCTURE AND FUNCTION	290	2004	Article	10.1247/csf.29.91
11	Briet, M. et al.	Arterial stiffness and pulse pressure in CKD and ESRD	KIDNEY INTERNATIONAL	287	2012	Review	10.1038/ki.2012.131
12	Zhou, XJ. et al.	The aging kidney	KIDNEY INTERNATIONAL	280	2008	Review	10.1038/ki.2008.319
13	Hommos, MS. et al.	Structural and Functional Changes in Human Kidneys with Healthy Aging	JOURNAL OF THE AMERICAN SOCIETY OF NEPHROLOGY	272	2017	Review	10.1681/ASN.2017040421
14	Denic, A. et al.	The Substantial Loss of Nephrons in Healthy Human Kidneys with Aging	JOURNAL OF THE AMERICAN SOCIETY OF NEPHROLOGY	269	2017	Article	10.1681/ASN.2016020154
15	Melk, A. et al.	Expression of p16INK4a and other cell cycle regulator and senescence associated genes in aging human kidney	KIDNEY INTERNATIONAL	258	2004	Article	10.1111/j.1523-1755.2004.00438.x

### 3.7 Hotspot and frontier analysis

Through a clustering analysis of keywords extracted from 3,082 publications, this study identified 82 high-frequency keywords (occurring more than 15 times) and constructed a keyword co-occurrence network (**Figure 8A**). In this network, node size represents keyword frequency, while the connecting lines visualize the co-occurrence strength between terms. Based on varying research focuses, these keywords are categorized into six core clusters that collectively outline the knowledge map of the kidney aging field. The dark blue cluster focuses on aging-related impacts and diseases, primarily exploring consequences such as kidney function, end-stage renal disease, and cardiovascular complications. The green cluster emphasizes factors associated with chronic kidney disease, such as obesity, hypertension, and age-related macular degeneration. The red cluster addresses biological effects induced by cellular senescence, involving acute kidney injury, oxidative stress, and renal aging. The yellow cluster centers on cellular physiology and organic changes, covering key processes such as autophagy, mitochondrial function, and ischemia. The purple cluster emphasizes renal pathological classifications, involving specific alterations like kidney fibrosis, diabetic nephropathy, and DNA damage. Finally, the light blue cluster focuses on tumor-related cellular behavior and genetic analysis.

**Figure 8:**
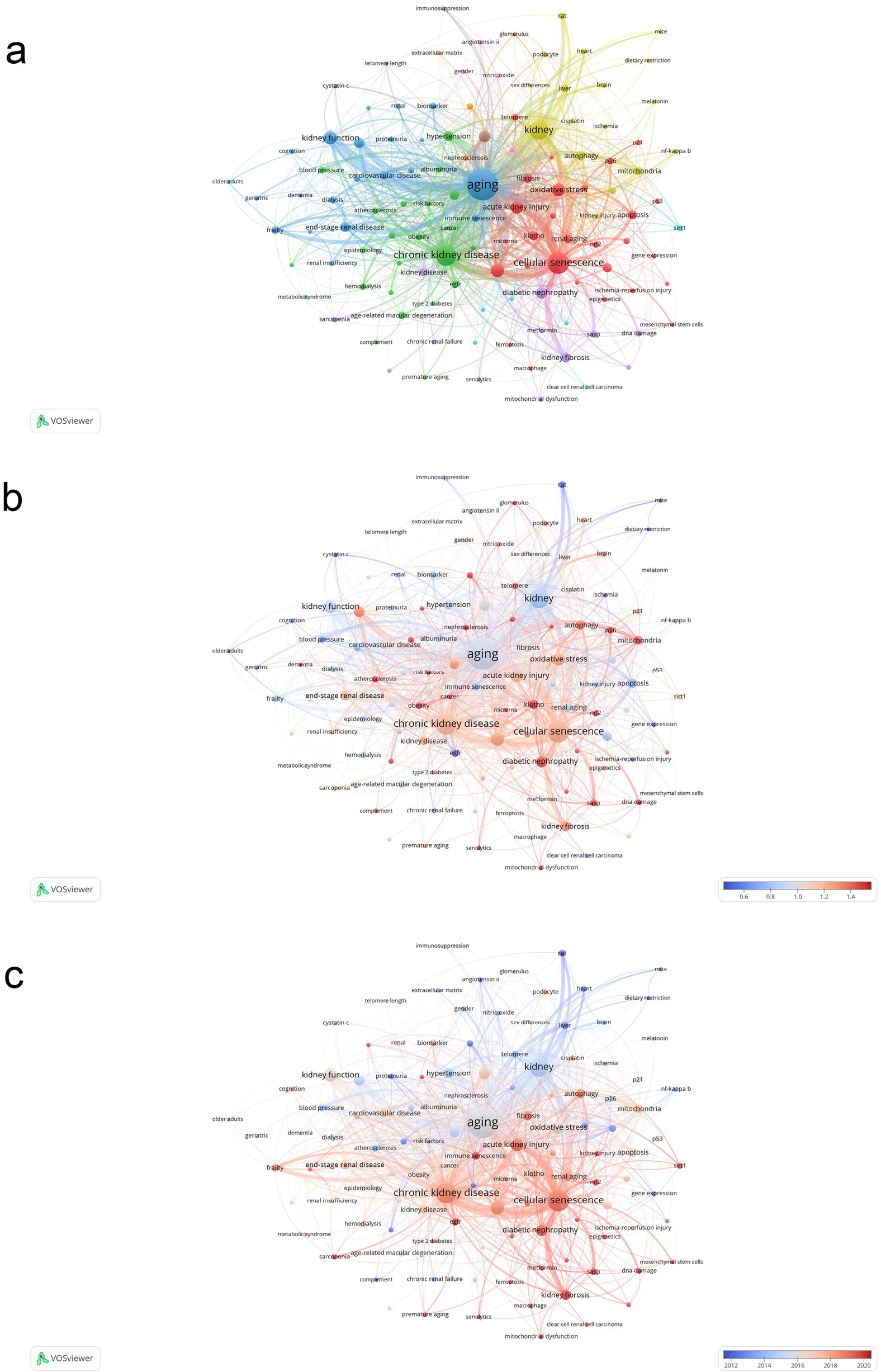
Keyword co-occurrence, citation intensity, and temporal evolution in kidney aging research. **(A)** Keyword co-occurrence network: distinct colors represent thematic clusters, with connecting lines illustrating the co-occurrence relationships between terms. **(B)** Average normalized citation counts: displayed using a color gradient from blue (low citation frequency) to red (high citation frequency) to indicate citation intensity. **(C)** Visualization of average publication years: a color-coded gradient from blue to red maps the temporal progression of research hotspots, reflecting the evolution from early clinical studies to recent frontier mechanisms.

**Table 6** summarizes the top 20 core keywords by frequency. Beyond “Aging” (825 occurrences) and “Cellular Senescence” (330 occurrences), terms such as “Chronic Kidney Disease” (306 occurrences), “Kidney” (261 occurrences), “Kidney Function” (107 occurrences), and “Inflammation” (103 occurrences) also exhibit high co-occurrence heat, establishing them as cornerstones in the kidney aging research framework. Furthermore, analyzing the average normalized citations of keywords (**Figure 8B**) helps identify the most influential trends; the color gradients map the citation intensity. The results clearly demonstrate that “Aging” is not only the most frequent foundational concept but also ranks first in normalized citations, further confirming that aging-centered mechanistic exploration remains a multidisciplinary focus and research highlight. Through the visualization of the average publication year (**Figure 8C**), this study delineates the temporal shift in research focus within the kidney aging field. Among core keywords, the average publication years for “Kidney” and “Aging” are concentrated around 2015 and 2016, respectively, while other high-frequency terms show higher usage between 2016 and 2020. The evolution of keywords clearly records the transition in disciplinary perspectives: from a focus on “Proteinuria” and “Angiotensin II” in 2012, to “Hypertension” and “Chronic renal failure” in 2015, followed by a surge in “Aging,” “End-stage renal disease,” and “Apoptosis” between 2016 and 2017. Since 2020, the research scope has further expanded into frontier mechanisms such as “Kidney fibrosis,” “Immune senescence,” and “NRF2.”

**Table 6:** Top 20 keywords in kidney and cell senescence research.

Rank	Keyword	Occurrences	Total link strength	Rank	Keyword	Occurrences	Total link strength
1	aging	825	1713	11	gfr	64	152
2	cellular senescence	330	707	12	hypertension	56	160
3	chronic kidney disease	306	743	13	end-stage renal disease	53	140
4	kidney	261	596	14	kidney fibrosis	53	119
5	kidney function	107	197	15	diabetes	50	158
6	inflammation	103	306	16	fibrosis	46	118
7	oxidative stress	101	292	17	autophagy	45	131
8	acute kidney injury	92	241	18	kidney aging	45	64
9	kidney transplantation	78	162	19	klotho	45	137
10	diabetic nephropathy	74	142	20	apoptosis	40	106

Citation Burst Analysis is a critical tool for identifying emerging research trends within a specific timeframe. As shown in **Figure 9A**, this study identified 25 burst keywords with the highest academic impact, among which “Cellular senescence” ranked first with a burst strength of 15.26, followed by “Renal fibrosis” (10.12) and “Secretory phenotype” (9.05). Currently, core keywords including “Cellular senescence,” “Renal fibrosis,” “Mitochondrial dysfunction” (7.31), “Functional changes” (5.76), “Diabetic kidney disease” (5.73), and “Inflammation” (4.91) maintain a continuous surge in citations, constituting the active frontline of current research. To further reveal the intrinsic connections between keywords, this study performed Multiple Correspondence Analysis (MCA) using the FactoMineR package (**Figure 9B**). This integrated map spatializes the similarities between terms using the dim1 and dim2 axes, where proximity represents higher similarity, making the keyword clustering logic more intuitive. The analysis shows that the top 50 keywords can be divided into four strategic quadrants: the upper-right quadrant (Quadrant I) corresponds to “motor themes” driving the field’s development; the upper-left quadrant (Quadrant II) represents highly specialized “niche themes”; the lower-left quadrant (Quadrant III) contains “emerging or declining themes”; and the lower-right quadrant (Quadrant IV) is locked as the “basic themes” of the field. Furthermore, MCA clustering revealed two primary research clusters, with the red cluster focusing on microscopic mechanistic exploration at the cellular and molecular levels, and the blue cluster focusing on macroscopic clinical disease research. Through a longitudinal analysis of keyword evolution, **Figure 9C** delineates the shifting paradigms within the kidney aging field from 2006 to 2024. The research trajectory initiated with a focus on macroscopic clinical phenotypes and comorbidities, as evidenced by early-stage keywords such as “diabetes mellitus,” “renal disease,” and “hypertension,” which dominated the landscape prior to 2015. Between 2016 and 2019, the field underwent a foundational transition toward mechanistic exploration, where “aging” emerged as the central pillar of research with the highest term frequency, accompanied by the rising prominence of “oxidative stress,” “autophagy,” and “inflammation”. The most recent period, spanning 2020 to 2025, signifies a transition into a frontier phase characterized by cellular precision and interdisciplinary integration. High-frequency terms such as “cellular senescence,” “renal tubular epithelial cells,” and “mitophagy” indicate a granular focus on cell-specific fate and organelle-level dysfunction. Notably, the emergence of “machine learning,” “ferroptosis,” and “mendelian randomization” as the most recent trend topics in 2025 suggests that the field is increasingly defined by the convergence of data-driven computational methodologies and novel metabolic regulation mechanisms. This evolution from descriptive clinical observation to integrated, multi-disciplinary mechanistic inquiry underscores the current frontier in kidney aging research. Combined with the trend evolution analysis in **Figure 9D**, it is evident that the research focus in the field of kidney aging is accelerating toward interdisciplinary directions such as “Machine learning,” “Ferroptosis,” and “Mendelian randomization,” signaling that the field has entered a new stage where data-driven approaches and metabolic regulation run in parallel.

**Figure 9:**
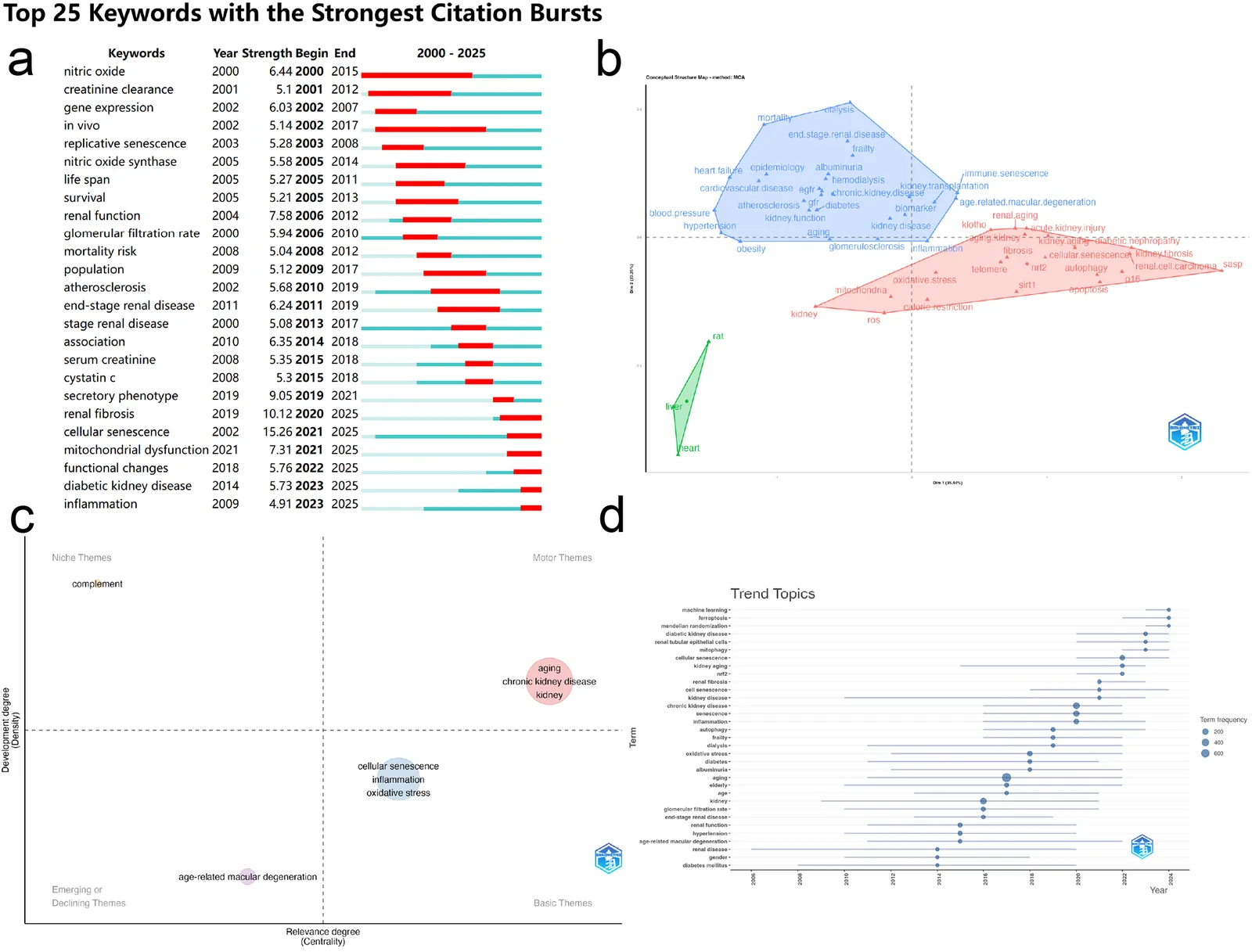
Citation bursts, thematic mapping, and trend evolution in kidney aging research. **(A)** Top 25 keywords with the strongest citation bursts: identifying key research frontiers in the field. **(B)** Multiple Correspondence Analysis (MCA) map: dimensions 1 and 2 (Dim1 and Dim2) represent the principal components derived from MCA, with values in parentheses indicating the cumulative percentage of explained variance. The map categorizes keywords into four strategic quadrants, effectively distinguishing between microscopic molecular mechanisms and macroscopic clinical research. **(C)** Thematic map: research themes are plotted based on density (y-axis) and centrality (x-axis), where closer proximity between keywords within a cluster indicates higher semantic similarity. **(D)** Trend topics visualization: the y-axis lists core keywords, while the x-axis represents the publication year. The size of the points indicates term frequency, tracing the temporal progression and shift of research hotspots over time.

To deepen the trend analysis, this study divided keywords into three stages: the Basic period (pre-2010), the Median period (2010–2018), and the Hotspot period (2019–2025). Principal Component Analysis (PCA) results (**Figure 10A**) showed a significant spatial separation between the Basic group and the other two stages. Although the Median and Hotspot groups exhibited some overlap, they maintained clear boundaries. This clustering characteristic provides strong evidence of a significant qualitative shift in keywords over time, confirming the scientific validity of the stage division. Subsequently, a popularity heat analysis using the Random Forest method (**Figure 10B**) highlighted core frontiers from the past five years. These include “Cellular senescence,” “COVID-19,” “Mitochondrial,” “Kidney fibrosis,” “DNA damaging,” “Sarcopenia,” and “NRF2.” Furthermore, the evolutionary trajectory (**Figure 10C**) spans from the earliest appearance of “Expression” in 2000 to the establishment of “Chronic kidney disease” as a core, persistently active theme in 2005. It concludes with the emergence of keywords such as “Signaling pathway,” “Breast cancer cells,” and “Cardiac fibrosis” in 2025. This progression fully illustrates the shift in kidney aging research from single-gene expression to complex signaling networks and interdisciplinary pathological correlations. These dynamic observations not only enhance our understanding of past research but also provide a data-driven foundation for identifying future scientific frontiers.

**Figure 10:**
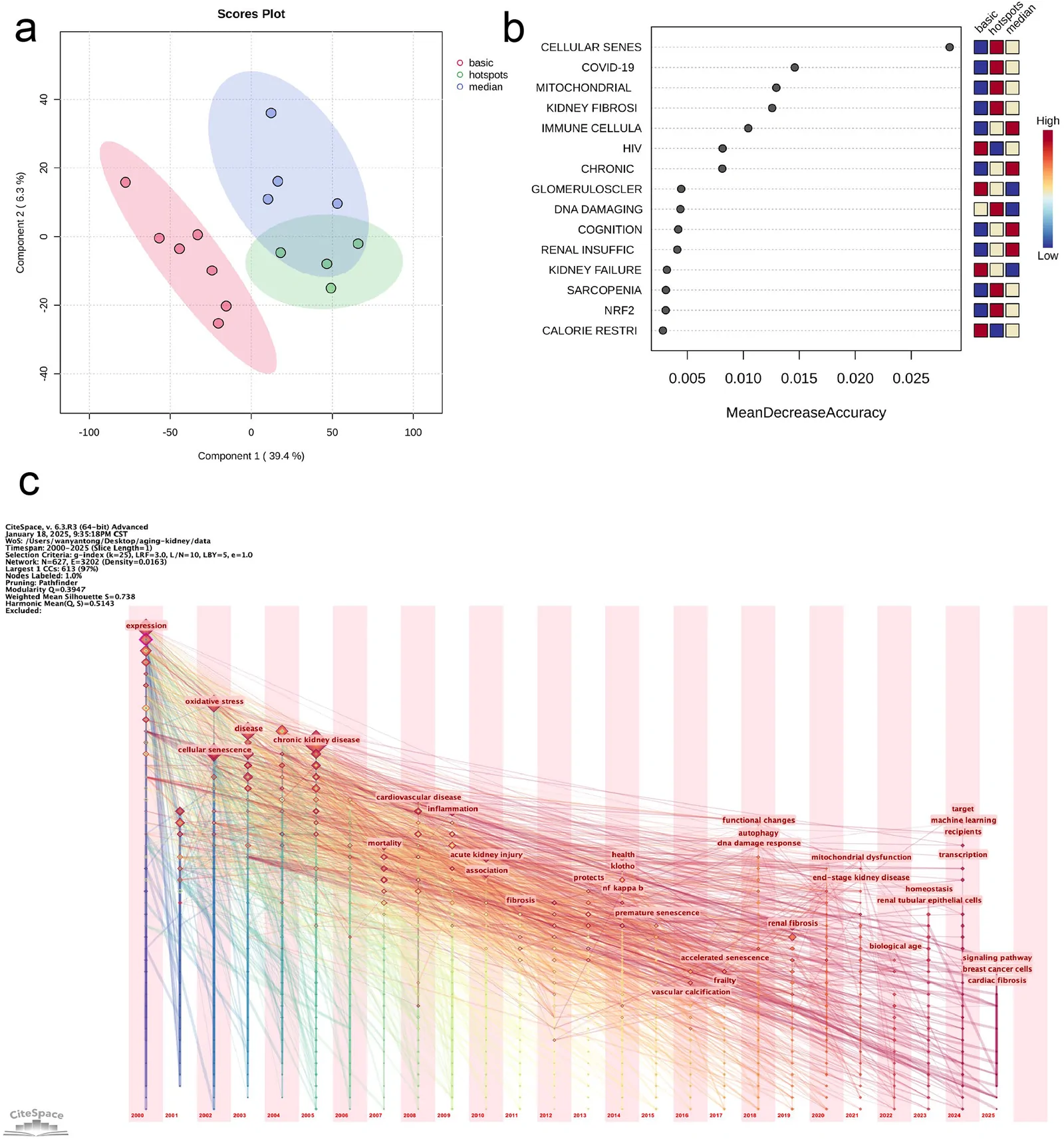
Temporal stage division, popularity heat, and evolutionary trajectory of research keywords in kidney aging. **(A)** Principal Component Analysis (PCA) scores plot: keywords are categorized into three distinct chronological periods represented by colored ellipses, with the distance between nodes indicating the strength of their association within each cluster. **(B)** Random Forest-based importance analysis: identifying core keywords in kidney aging research; the heatmap on the right illustrates the temporal dynamics of term popularity across three stages (basic, hotspots, and median), with the color gradient (from blue to red) representing increasing prevalence. **(c)** Evolutionary trajectory visualization: generated via CiteSpace, this network traces the dynamic transition and complex associations of research keywords over time, with line and node density reflecting the integration of knowledge structures and the emergence of new research frontiers.

## 4 Discussion

### 4.1 Overall analysis

This bibliometric analysis of 2,191 WoSCC papers (2000–2025) reveals rapid growth in kidney aging research, with annual publications rising from under 100 before 2018 to 320 by 2025, alongside citations reaching a high of 8,577 in 2025. The United States dominates globally with 587 papers and 25,229 citations, followed by China (399 papers; 7,734 citations). The Mayo Clinic leads institutional metrics (44 papers; 3,651 citations), ahead of the Chinese PLA General Hospital (38 papers), with US centers claiming six of the top ten spots. Across 51 journals, the *American Journal of Physiology-Renal Physiology* leads in volume, while *JASN* and *Kidney International* each exceed 4,000 co-citations. Among 16,545 authors, Xiang-Mei Chen (40 papers) and Anette Melk (379 citations) are top contributors; however, Lotka’s Law indicates a developing field, as over 75% of researchers published only once. Thematically spanning 18 reference clusters, focus has shifted from early allograft nephropathy toward intrinsic cellular mechanisms. Current hotspots and citation surges center on diabetic kidney disease, cell cycle arrest, and targeted clearance of senescent cells, exemplified by Hickson LJ’s clinical study and Eoin D. O’Sullivan’s highly cited 2017 review. Ultimately, these trends highlight a critical transitional phase in nephrology driven by a massive influx of new researchers and shifting molecular paradigms. As the field continues to mature, translating mechanistic insights of cellular senescence into targeted clinical therapies will undoubtedly remain the paramount scientific objective.

### 4.2 Hotspots and trends

From 2000 to 2025, mechanistic inquiries into renal aging have progressively transitioned from descriptive histomorphological observations toward precision molecular networks governing cellular fate decisions, organelle-level quality control, and systemic inter-organ metabolic crosstalk (12, 23). Cellular senescence operates as the quintessential pathobiological hub connecting chronic kidney disease (CKD), acute kidney injury (AKI), diabetic kidney disease (DKD), and progressive renal fibrosis (11, 13, 24). Our bibliometric synthesis delineates this paradigm shift across multiple dimensional tiers: early 21st-century investigations focused predominantly on extracellular matrix expansion, chronic allograft nephropathy, and vascular calcification (25, 26), whereas the landscape after 2018 underwent an exponential inflection point characterized by the molecular deconstruction of senescence-associated secretory phenotypes (SASP), immunosenescence, programmed cell death crosstalk, and organelle bioenergetics (9, 27, 28). Most recently, high-dimensional algorithmic screening and genetic causal inference—such as machine learning and Mendelian randomization—alongside microenvironmental gut–kidney interactions have defined the modern research frontier (29–32). Based on high-frequency keyword co-occurrence clusters (**Table 6**, **Figure 8A**), citation burst detection (**Figure 9A**), and co-citation topological networks (**Table 5**, **Figure 7**), this discussion provides an integrated, data-grounded synthesis across three consolidated core domains.

#### 4.2.1 Core molecular cascades, actionable targets, and senotherapeutic strategies

Bibliometric metrics firmly establish that cell cycle arrest machinery constitutes the primary regulatory engine driving renal functional decline. In our keyword burst analysis, “cellular senescence” ranked first with the highest academic burst strength of 15.26 (active from 2021 to 2025), closely partnered by “renal fibrosis” (burst strength = 10.12, 2020–2025) and “secretory phenotype” (burst strength = 9.05, 2019–2021) (**Figure 9A**). Moreover, “aging” (825 occurrences) and “cellular senescence” (330 occurrences) serve as the foundation of the keyword co-occurrence network (**Table 6**). Mechanistically, persistent cell cycle arrest in parenchymal cells—predominantly renal proximal tubular epithelial cells (PTECs), podocytes, and vascular endothelia—is coordinated via the classic p53/p21^CIP1^ and p16^INK4a^/Rb signaling cascades (33). Notably, although both pathways execute cell cycle withdrawal, their pathophysiological kinetics exhibit distinct temporal dynamics: p21 mediates early, reversible cellular stress responses following acute tubular or glomerular insults, maintained by hypomethylation of the p21 gene promoter (34). In contrast, p16^INK4a^ marks an irreversible, chronic senescent state that solidifies progressive tissue remodeling. As demonstrated by da Silva et al. (2025), this kinetic trajectory defines the molecular “tipping point” between physiological aging and irreversible functional collapse: middle-aged kidneys exhibit early upregulation of p21 and p16 alongside oxidative stress prior to overt functional impairment, whereas aged kidneys progress to widespread p53, COX-2, NF-\κB p65 activation, and destructive SASP cascades. The translational feasibility of senescent cell elimination has been validated by seminal co-citation bursts. Darren J. Baker’s milestone work demonstrating that clearing p16^INK4a^ -positive cells extends healthspan demonstrated profound academic influence (burst strength = 21.04, **Figure 7C**), while the landmark clinical trial by Hickson et al. (2019) ranks first among the top 15 most co-cited references in our dataset (758 citations, **Table 5**). Hickson et al. established that senolytic cocktails combining dasatinib (a broad-spectrum tyrosine kinase inhibitor) and quercetin (a natural flavonoid targeting BCL-2/BCL-xL pro-survival networks) successfully reduce senescent cell burden and attenuate pro-inflammatory SASP expression in patients with diabetic nephropathy (35). In addition, targeted clearance of p16-positive cells in preclinical transgenic models (such as INK-ATTAC systems) restores renal ATP reserves, reprograms aberrant glycolysis, normalizes mitochondrial oxidative metabolism via AMPK/mTOR pathways, and alleviates interstitial matrix deposition (36, 37). Beyond small-molecule senolytics like navitoclax (ABT-263) and fisetin (14, 15), emerging senomorphic strategies aim to uncouple the SASP secretome by selectively targeting NF-κB, p38 MAPK, and cGAS-STING signaling without inducing cytotoxic cell lysis, thereby mitigating secondary paracrine senescence in adjacent functional nephrons (38, 39). Concurrently, our bibliometric atlas reveals an intimate mechanistic nexus connecting cellular senescence, mitochondrial collapse, and regulated cell death pathways. Keyword burst detection identified “mitochondrial dysfunction” (burst strength = 7.31, 2021–2025, **Figure 9A**) and “oxidative stress” (101 occurrences, total link strength = 292, **Table 6**) as persistent interaction hubs with cell death modalities (**Figure 8A**). Renal tubular epithelial cells possess immense baseline metabolic demands predominantly powered by mitochondrial fatty acid β-oxidation and oxidative phosphorylation (40). During chronological aging and sustained pathological stress, defective mitochondrial quality control (MQC)—manifested as down-regulated GLIS1/PGC-1α biogenesis, impaired PINK1/Parkin-mediated mitophagy, and NOX4-Nrf2 redox uncoupling—leads to “autophagic stagnation” and excessive accumulation of damaged, reactive oxygen species (ROS)-generating mitochondria (41–43). Furthermore, aberrant mitochondrial calcium influx mediated by upregulation of the mitochondrial calcium uniporter (MCU) or AT1R/PKA/CREB-dependent TRPC3 activation directly exacerbates mitochondrial permeability transition and ROS bursts, propelling parenchymal senescence (44, 45). Damaged mitochondria subsequently release mitochondrial DNA (mtDNA) into the cytosol, which triggers the cGAS-STING inflammatory cascade to amplify the SASP secretome and propagate sterile tubulointerstitial inflammation (46).

Crucially, keyword trend analysis identifies “ferroptosis”—an iron-dependent non-apoptotic cell death mechanism driven by lethal lipid peroxidation—as one of the most prominent recent hotspots (**Figure 9D**) (47). In the aging kidney, iron accumulation, system Xc^-^/GSH/GPX4 axis exhaustion, and ACSL4-mediated polyunsaturated fatty acid enrichment establish a heightened vulnerability toward ferroptotic cell death (48, 49). Ferroptosis acts as a preemptive trigger in AKI and forms a pathological bridge to CKD progression by releasing damage-associated molecular patterns (DAMPs) and activating innate immune cascades (50, 51). Simultaneously, selective degradation of ferritin via ferritinophagy promotes intracellular free iron release, coordinating an intricate feedback loop between autophagy, ferroptosis, and senescence (52–54). Parallel regulatory cascades, including the FSP1-CoQ10-NAD(P)H and GCH1-BH4 systems, represent promising GPX4-independent therapeutic targets to curb lipid peroxidation and renal fibrosis (55, 56). A wide spectrum of pharmacological interventions has shown striking efficacy: the endogenous longevity factor Klotho restores mitochondrial mass through LKB1-AMPK-PGC-1α axis reactivation while repressing profibrotic Wnt/β-catenin and RAS signaling (57, 58); small-molecule β-catenin destabilizers (KYA1797K) (59), soluble epoxide hydrolase (sEH) inhibitors (60), MCU antagonists and repurposed metabolic agents (e.g., empagliflozin, fenofibrate) (61) effectively preserve organelle homeostasis. Furthermore, bioactive traditional Chinese medicine compounds, such as formononetin (suppressing Smad3/ATF3/SLC7A11 signaling) and Nrf2 activators, offer robust multi-target protection against ferroptosis-associated tubular senescence and interstitial fibrosis (23).

#### 4.2.2 The gut-kidney-immune axis: microbial metabolites in senescence acceleration and intervention

A transformative paradigm shift emerging in nephrology is the realization that renal cellular senescence is not an isolated intrarenal degenerative process, but rather deeply anchored within a systemic microbiota-gut-kidney-immune communication axis (62, 63). While early bibliometric clusters concentrated predominantly on local glomerular hemodynamics and isolated tubulointerstitial scarring (**Figures 7A**, **8A**), current trend analyses underscore the indispensable contribution of intestinal dysbiosis, gut mucosal barrier disruption, and circulating microbial oncometabolites in accelerating biological aging of the kidney (64, 65). This conceptual expansion directly aligns with our bibliometric findings, where “inflammation” exhibits a sustained citation burst (burst strength = 4.91, active 2023–2025, **Figure 9A**), and keywords related to immune dysfunction show high temporal centrality in CiteSpace network projections (**Figure 10B, C**).

The pathophysiological continuum of the gut-kidney axis operates as a bidirectional, self-perpetuating vicious cycle across biological barriers (32). Under physiological conditions, an intact intestinal epithelial layer and a symbiotic commensal microbiome maintain host metabolic and immune homeostasis (66, 67). However, with advancing age and the progressive decline of glomerular filtration rate (GFR) (**Table 6**, occurrence = 64), urea and other metabolic wastes diffuse into the gastrointestinal tract (5, 68). Urease-possessing bacterial families expand dramatically, elevating luminal pH, degrading protective mucin layers, and disassembling tight junction complexes such as ZO-1, occludin, and claudins (31, 32). This compromised “leaky gut” facilitates the massive translocation of microbial-derived uremic toxins—predominantly indoxyl sulfate (IS), p-cresyl sulfate (PCS), and trimethylamine N-oxide (TMAO)—as well as bacterial endotoxins such as lipopolysaccharide (LPS) directly into the systemic circulation (62). Upon entering the renal microcirculation, circulating IS and PCS are avidly taken up by proximal tubular epithelial cells via organic anion transporters (OAT1/3). Intracellular accumulation of these toxic metabolites unleashes severe oxidative stress by upregulating NADPH oxidase 4 (NOX4) and disrupting mitochondrial electron transport chains. This uremic insult induces telomeric and non-telomeric DNA double-strand breaks, triggering persistent activation of the ATM/ATR-p53-p21 and p16-Rb cascades, thereby driving PTECs and glomerular podocytes into irreversible premature cellular senescence (69, 70). Senescent parenchymal cells subsequently hypersecrete copious SASP factors—including TGF-β1, IL-6, IL-1β, TNF-α, and MCP-1/CCL2—which stimulate quiescent interstitial fibroblasts into α-SMA-positive myofibroblasts, directly driving the citation-burst active term “renal fibrosis” (burst strength = 10.12). Furthermore, gut-derived TMAO directly fuels intrarenal lipid accumulation, exacerbates ferroptotic lipid peroxidation, and activates the NLRP3 inflammasome, accelerating microvascular rarefaction and glomerulosclerosis (71). Crucially, modern multi-omics investigations reveal that the gut-kidney axis coordinates a state of profound immune dysregulation that perpetuates senescence (72, 73). The leakage of bacterial endotoxins and microbial indoles engages the aryl hydrocarbon receptor (AHR) and Toll-like receptors (TLR4/MyD88/NF-κB), driving an imbalance in mucosal and systemic innate lymphoid cells (ILCs). The trans-tissue trafficking of inflammatory ILC subsets, coupled with an expanded Th17-to-regulatory T cell (Treg) ratio, exacerbates tubulointerstitial infiltration (74). Concurrently, persistent uremic and endotoxic stimulation accelerates systemic T-cell senescence and thymic involution, establishing secondary immunodeficiency related to kidney disease (SIDKD). This age-associated immune exhaustion blunts the physiological immune surveillance and phagocytic clearance of intrarenal senescent cells, fueling chronic low-grade “inflammaging”. Conversely, beneficial microbial fermentation metabolites, specifically short-chain fatty acids (SCFAs: acetate, propionate, and butyrate), serve as indispensable endogenous brakes against renal aging and fibrogenesis (75, 76). SCFAs bind to G-protein-coupled receptors (GPR41, GPR43, and GPR109A) on renal parenchymal and immune cells, blunting NF-κB phosphorylation, activating Nrf2-mediated antioxidant transcription, and sustaining mitochondrial respiratory competency. In addition, butyrate functions as an endogenous histone deacetylase (HDAC) inhibitor, epigenetically suppressing profibrotic gene transcription and downregulating SASP expression. In the aging or diseased kidney, the depletion of SCFA-producing commensals (such as Faecalibacterium prausnitzii, Roseburia, and Bifidobacterium) removes this protective constraint, accelerating both local and systemic senescence (74). Targeting the microbiota-gut-kidney-immune interface has therefore become a pivotal therapeutic hotspot with vast translational potential. Oral spherical carbon adsorbents such as AST-120 trap indole and p-cresol precursors directly in the intestinal lumen, substantially lowering circulating uremic toxin concentrations, mitigating intrarenal oxidative stress, and attenuating tubular senescence cascades. Simultaneously, microbiome ecological modulators—including targeted prebiotics (such as inulin and resistant starch), next-generation probiotics (such as Akkermansia muciniphila, Lactobacillus reuteri, and Bifidobacterium animalis), synbiotics, and postbiotic SCFAs—reinforce intestinal tight junction integrity, suppress systemic endotoxin leakage, downregulate renal TLR4/NF-κB signaling, and restore immune equilibrium. Furthermore, reconstituting complete, healthy gut microbial consortia via fecal microbiota transplantation (FMT) has shown profound preclinical and early clinical efficacy across CKD and renal transplantation models by inhibiting intrarenal renin-angiotensin system (RAS) activation, re-establishing Treg/Th17 balance, and reversing uremic toxin-induced premature senescence (77). Additionally, holistic traditional Chinese medicine preparations, such as Uremic Clearance Granules (UCG), remodel gut microbiota composition by enriching B. animalis and elevating bioactive sophocarpine, thereby repairing the intestinal mucosal barrier, rebalancing Th17/Treg immunity, and providing multi-pathway therapeutic protection against chronic renal failure progression.

#### 4.2.3 Data-driven paradigms and frontier perspectives for translational nephrology

Our longitudinal and strategic mapping reveals that kidney aging research is advancing into an interdisciplinary era characterized by the convergence of computational intelligence, genetic causal modeling, and cell-targeted nanomedicine. The Trend Topics analysis (**Figure 9D**) and Random Forest feature importance ranking (**Figure 10B**) demonstrate that “machine learning” and “Mendelian randomization” have surfaced in 2024–2025 as the latest algorithmic frontiers driving the discipline. Furthermore, the CiteSpace evolutionary trajectory map (**Figure 10C**) traces the disciplinary progression from single-gene exploration (“expression” in 2000) to complex multi-target signaling networks (“signaling pathway” in 2025) and systemic multi-organ pathologies (“cardiac fibrosis” in 2025). The deployment of machine learning algorithms—including random forest, support vector machines, deep neural networks, and LASSO regression—is profoundly transforming target discovery and disease stratification. When combined with high-dimensional single-cell RNA sequencing (scRNA-seq), single-nucleus ATAC-seq, and spatial transcriptomics, machine learning empowers researchers to deconvolve cellular senescence heterogeneity across distinct nephron segments with unprecedented resolution. Rather than treating renal senescence as a uniform phenomenon, computational clustering reveals distinct cell-type-specific senescence trajectories: proximal tubular cells predominantly exhibit metabolic reprogramming, autophagic stalling, and ferroptosis vulnerability; podocytes display early *p21*-driven hypertrophic arrest, effacement, and mechanical detachment; and peritubular capillary endothelial cells undergo primary barrier breakdown and SASP amplification. These algorithmic pipelines allow the precise identification of segment-specific senescence biomarkers and the prioritization of druggable molecular switches governing the tipping point of irreversible fibrosis. Parallel to machine learning, Mendelian randomization (MR) has emerged as a cornerstone methodology to establish causal relationships in kidney aging and metabolic research. By utilizing single-nucleotide polymorphisms (SNPs) as instrumental variables from extensive genome-wide association studies (GWAS), MR overcomes the classical vulnerabilities of observational epidemiology, such as reverse causality and unmeasured confounding. Recent MR investigations have provided compelling genetic causal evidence confirming that genetically predicted gut microbiota taxa, circulating microbial metabolites (such as TMAO, indoles, and SCFA ratios), and ferroptosis-associated genetic variants exert direct causal effects on estimated GFR decline, CKD susceptibility, and diabetic kidney disease progression. Integrating bidirectional, multivariable, and two-step MR approaches will further map the directional causal hierarchy connecting gut microbiome composition, circulating metabolome alterations, programmed cell death activation, and renal cellular senescence, providing high-confidence targets for subsequent preclinical and clinical validation. Synthesizing the Multiple Correspondence Analysis (MCA) strategic quadrants (**Figure 9B**) and the dual-map overlay of knowledge flow (**Figure 5C**), future research must focus on bridging the remaining disconnect between microscopic molecular biology and macroscopic clinical translation. At the subcellular level, future studies should leverage spatial multi-omics to dissect how mitochondrial dysfunction, mitophagy stalling, and telomere DNA damage interact in distinct spatial microenvironments of the aging cortex and medulla (78). To overcome the major translational bottleneck of current senotherapeutics, iron chelators, and microbiome modulators—namely systemic off-target toxicity and poor intrarenal bioavailability—future translational efforts must exploit engineered mesenchymal stem cell-derived extracellular vesicles (MSC-EVs) delivering protective mitochondrial transcription factor A (TFAM), folate- or peptide-functionalized nanoparticles (79), and targeted exosome carriers to achieve cell-specific cargo delivery directly to injured tubular epithelial cells or podocytes. Finally, the emergence of terms such as “cardiac fibrosis” in our 2025 trajectory map (**Figure 10C**) emphasizes that renal senescence is deeply intertwined with systemic organ decline (80). Deciphering the shared molecular interconnections within the gut-heart-kidney-immune axis will provide a systemic foundation for multi-organ anti-aging regimens, transforming clinical nephrology from late-stage symptom management into early, data-driven, precision geroprotection.

## 5 Limitations

This study has certain limitations. First, data was sourced exclusively from the WoSCC andv Scopus database and restricted to English, potentially omitting regional literature. Second, varying publication quality may introduce bias. Third, clustering algorithms rely on extracted terms, meaning synonymous terms might not be grouped perfectly. These limitations should be considered when interpreting the conclusions.

## 6 Conclusion

Despite its limitations, this study maps the intellectual structure of kidney and cellular senescence research from 2000 to 2025. This comprehensive bibliometric analysis of 2,191 papers and 16,545 researchers reveals that kidney aging research has entered a phase of exponential growth, reaching its historical peak in 2025. While the United States and China maintain dominant positions in publication volume and collaborative networks, the field’s academic gravity is anchored by core journals such as the *Journal of the American Society of Nephrology* and the foundational leadership of pioneering scholars like Xiang-Mei Chen and Anette Melk. Beyond statistical growth, the analysis identifies a profound paradigm shift that redefines the future of the field, moving from macroscopic clinical phenotypes such as hypertension and diabetes toward precise cellular fate regulation. The persistent focus on “cellular senescence,” “mitophagy,” and “renal fibrosis” suggests that targeting organelle-level dysfunction and molecular signaling networks has become the cornerstone of therapeutic exploration. Furthermore, the evolution of research hotspots indicates that kidney aging is increasingly viewed through a systemic, interdisciplinary lens, integrating “immune senescence” and metabolic regulation with complex pathological correlations. Most significantly, the rapid rise of “machine learning,” “ferroptosis,” and “Mendelian randomization” marks a transition from descriptive observation to a data-driven frontier. This convergence of computational intelligence and novel metabolic pathways offers transformative potential for identifying robust biomarkers and early intervention targets, signaling a new era of personalized precision medicine for age-related renal diseases.

## Data Availability

The original contributions presented in the study are included in the article/**Supplementary Material**. Further inquiries can be directed to the corresponding authors.
